# Robust Kalman Filter Aided GEO/IGSO/GPS Raw-PPP/INS Tight Integration

**DOI:** 10.3390/s19020417

**Published:** 2019-01-21

**Authors:** Zhouzheng Gao, You Li, Yuan Zhuang, Honglei Yang, Yuanjin Pan, Hongping Zhang

**Affiliations:** 1 School of Land Science and Technology, China University of Geosciences Beijing, Beijing 100083, China; zhouzhenggao@126.com (Z.G.); yanghonglei226@126.com (H.Y.); 2 Shanxi Provincial Key Laboratory for Resources, Environment and Disaster Monitoring, Jinzhong 030600, China; 3 Department of Geomatics Engineering, University of Calgary, Calgary, AB T2N 1N4, Canada; 4 State Key Laboratory of Information Engineering in Surveying, Mapping and Remote Sensing, Wuhan University, Wuhan 430079, China; yjpan@whu.edu.cn; 5 GNSS Research Center, Wuhan University, Wuhan 430079, China; hpzhang@whu.edu.cn

**Keywords:** robust extended Kalman filter (R-EKF), geosynchronous Earth orbit (GEO) satellites, inclined geo-synchronous orbit (IGSO) satellites, precise point positioning (PPP), inertial navigation system (INS)

## Abstract

Reliable and continuous navigation solutions are essential for high-accuracy location-based services. Currently, the real-time kinematic (RTK) based Global Positioning System (GPS) is widely utilized to satisfy such requirements. However, RTK’s accuracy and continuity are limited by the insufficient number of the visible satellites and the increasing length of base-lines between reference-stations and rovers. Recently, benefiting from the development of precise point positioning (PPP) and BeiDou satellite navigation systems (BDS), the issues existing in GPS RTK can be mitigated by using GPS and BDS together. However, the visible satellite number of GPS + BDS may decrease in dynamic environments. Therefore, the inertial navigation system (INS) is adopted to bridge GPS + BDS PPP solutions during signal outage periods. Meanwhile, because the quality of BDS geosynchronous Earth orbit (GEO) satellites is much lower than that of inclined geo-synchronous orbit (IGSO) satellites, the predicted observation residual based robust extended Kalman filter (R-EKF) is adopted to adjust the weight of GEO and IGSO data. In this paper, the mathematical model of the R-EKF aided GEO/IGSO/GPS PPP/INS tight integration, which uses the raw observations of GPS + BDS, is presented. Then, the influences of GEO, IGSO, INS, and R-EKF on PPP are evaluated by processing land-borne vehicle data. Results indicate that (1) both GEO and IGSO can provide accuracy improvement on GPS PPP; however, the contribution of IGSO is much more visible than that of GEO; (2) PPP’s accuracy and stability can be further improved by using INS; (3) the R-EKF is helpful to adjust the weight of GEO and IGSO in the GEO/IGSO/GPS PPP/INS tight integration and provide significantly higher positioning accuracy.

## 1. Introduction

Precise point positioning (PPP) [1], which is based on the ionosphere-free combination of dual-frequency (L1: 1575.42 MHz and L2: 1227.6MHz) Global Positioning System (GPS) phase and code observations [2], was proposed two decades ago and has been adopted for static positioning applications [3,4,5,6]. This phenomenon is mainly due to the two advantages of PPP. First, PPP has the capability to efficaciously provide centimeter-level or even millimeter-level absolute positioning solutions for static applications under open-sky environments, such as earthquake monitoring [3,4], crust movement measuring [5], and water vapor prediction [6]. Moreover, PPP does not need base stations, which makes it possible to obtain high-accuracy PPP solutions at any place as long as there are enough available GPS observations. This is because PPP uses precise error correction models and precise satellite orbit/clock products to mitigate observing errors and estimates the incomplete modeled errors as parameters [1,7]. Such data processing methods can drastically overcome the challenge that the positioning error may become large along with the increasing base-line, which is an issue inherent to real-time kinematic (RTK) systems [8].

**Figure 1:**
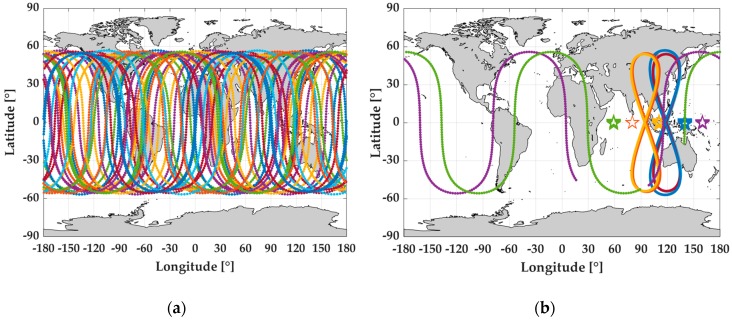
Sky-plots of GPS satellites (MEO) (**a**) and BDS GEO/IGSO/MEO regional satellites (**b**). MEO is medium Earth orbit, BDS is BeiDou satellite navigation system, GEO is geostationary Earth orbit, and IGSO is inclined geosynchronous satellite orbit.

However, compared to PPP, RTK has been more widely applied in the precise positioning and navigation engineering practices, especially in dynamic applications, such as aerial photogrammetry and mobile mapping. The main reason for this fact is that the frequently decreased visible satellite number will lead to insufficient observations for PPP parameter estimation, and lower positioning accuracy and continuity [9,10]. In order to abate such shortcoming in PPP, the multi-constellation global navigation satellite systems (GNSS) data are utilized [11,12,13,14]. For example, in [11] and [14], data from the BeiDou satellite navigation system (BDS), GLObal NAvigation Satellite System (GLONASS), and Galileo are adopted to improve the GPS PPP solutions. In contrast to the other three GNSS systems (i.e., GPS, GLONASS, and Galileo adopt the medium Earth orbit (MEO) satellites constellation), BDS consists of three types of orbit-satellites, namely geostationary Earth orbit satellites (GEO), inclined geosynchronous satellite orbit satellites (IGSO), and MEO satellites [15]. Theoretically, the existence of GEO and IGSO can provide better satellite tracking and observing geometry structure for users [16], especially in China, because of their special locations. According to [17,18], BDS is China’s global navigation satellite system, which was built around 2000 to provide triple-frequency (B1I: 1561.098 MHz, B2I: 1207.140 MHz, and B3I: 1268.520 MHz) civil signals to satisfy the location service requirements of China. Before BDS-3’s open service online (i.e., before 27 December 2018), BDS only provided navigation and precise positioning service in the Asia–Pacific region with the operating satellite constellation of five GEOs, five IGSOs, and four MEOs, respectively [19]. Figure 1 shows the sky plot of the current GPS (Figure 1a) and BDS regional system (Figure 1b), which brings significant enhancement to increasing the total visible satellite number around China. As proven by the previous works, the use of BDS observations can increase visible satellite number [11,14,15] and upgrade GPS PPP performance significantly in terms of convergence speed, positioning accuracy, and continuity [14].

In dynamic applications, users have to frequently pass through avenues, flyovers, and tunnels, especially in urban environments. Under those conditions, satellite signals would be blocked. Accordingly, the visible satellite number may be reduced to under four, which is not sufficient for PPP computation, even when using GPS and BDS together [20,21]. In order to bridge PPP solutions during those signal outages, the inertial navigation system (INS) [22,23] is utilized. INS is an autonomous positioning technique and can provide continuous positioning solutions by processing the specific force measurements from triaxial accelerometers and the angular increments from triaxial gyroscopes [24,25]. Meanwhile, INS solutions are accurate in the short term and it is hardly influenced by surrounding environments [24]. Thus, PPP’s performance in challenging conditions can be bridged by adopting the integration of PPP and INS. As such, according to [26], two integration modes can be used, namely loose integration and tight integration. The tight integration mode is based on the raw GPS/BDS phase/code observations and the INS predicted phase/code values [20,21], while the loose integration mode is based on PPP position and velocity solutions and that of the INS updated ones [25,26]. Accordingly, the tight integration mode can also work in the case that the observed satellite number is inadequate for PPP’s calculation. Therefore, the tight integration model between GPS/BDS and INS is adopted in this paper.

Nevertheless, there are only four MEO satellites in BDS and the time in view is hours. Hence, usually only GEOs and IGSOs are visible in most of the dynamic applications. According to the BDS quality analysis works in [27,28,29], the qualities of BDS GEO, IGSO, and MEO are different from each other due to the different orbit accuracy, observation noise, and multipath. Particularly, the quality of GEO is proven to be several times lower than that of IGSO and MEO. However, usually the elevation dependent weight function [6] is used to present satellite quality. Such a method is not significantly rigorous for real observed data, especially for BDS. In this paper, the famous robust extended Kalman filter (R-EKF) [30,31] that is based on equivalent weight function is employed to reduce the impact of the inappropriate priori weight of BDS GEO. Besides, according to the works in [32], the un-differenced un-combined PPP (i.e., raw PPP) is more powerful than the ionosphere-free combination PPP (i.e., IF PPP). Therefore, the raw PPP mode is adopted in this paper. Compared to previous works, this paper’s contributions are: (1) The impacts of BDS GEO satellites and IGSO satellites on GPS + BDS PPP and GPS + BDS PPP/INS tight integration are analyzed and evaluated. (2) To discriminate the different qualities of BDS GEO and IGSO satellites, this paper introduces the equivalent weight function based R-EKF, instead of the empirical satellite elevation angle weight function based EKF, to distinguish the real contributions from IGSO and GEO in GEO/IGSO/GPS PPP/INS tight integration. To validate the proposed method, a set of land-borne vehicle GPS/BDS data and two groups of INS data were processed and analyzed.

The paper is organized as follows: The mathematical models of BDS GEO/IGSO enhanced PPP, INS update, and R-EKF based PPP/INS tight integration are described in Section 2. The tests and results are presented in Section 3, which is followed by the conclusions in Section 4.

## 2. Methods

**Figure 2:**
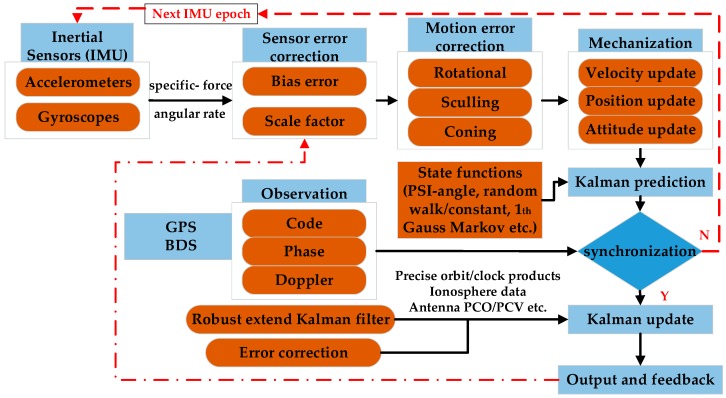
Algorithm structure of R-EKF aided GPS/BDS PPP/INS tight integration. R-EKF is robust extended Kalman filter, PPP is precise point positioning, INS is inertial navigation system, IMU is inertial measurement unit, and PCO/PCV is phase center offset/variation.

In this section, the mathematical models of the GPS/BDS raw PPP, INS mechanization, and R-EKF enhanced PPP/INS tight integration are described. As shown in Figure 2, INS provides position, velocity, and attitude for users by processing the specific force and angular rate measurements from inertial measurement units (IMU) in the INS mechanization [10] after the correction for sensors errors (e.g., IMU sensor biases and scale factors) and motion errors (e.g., rotational, sculling, and coning motion) [25]. Then, the EKF prediction is applied to provide the predicted covariance matrix according to the state functions and the initial covariance matrix. Afterwards, time synchronization between GPS/BDS observations and INS solutions is checked. If there is no visible GPS/BDS observation, it goes to the next IMU epoch. Otherwise, the EKF innovation is calculated by obtaining the difference between GPS/BDS observations (e.g., code, phase, and Doppler) and the INS predicted values; the INS predicted values are computed by using the INS updated position/velocity and the satellite position/velocity after considering the GPS/BDS related errors (e.g., antenna phase center offset/variation (PCO/PCV), ionosphere delay, troposphere delay, and earth rotation [33]). To distinguish the real impacts of BDS GEO and IGSO on PPP/INS tight integration, the R-EKF is utilized. Then, the corrections for position, velocity, attitude, and IMU biases and scale factors are estimated and fed back to compensate for IMU sensor errors in the next IMU epoch.

### 2.1. GEO/IGSO/GPS Raw PPP Model

In contrast to the conventional PPP model [1], the raw PPP mode has been proven to perform better in accelerating convergence speed and decreasing solution noise [32]. The basic observation models for un-differenced un-combined phase, code, and Doppler observations are

(1) $$P_{r,j}^{s}=\rho_{r}^{s}+c(t_{r}-t^{s})+c(d_{r,j}-d_{j}^{s})+T_{r}^{s}+I_{r,j}^{s}+\varepsilon_{P,j}$$

(2) $$L_{r,j}^{s}=\rho_{r}^{s}+c(t_{r}-t^{s})+c(b_{r,j}-b_{j}^{s})+T_{r}^{s}-I_{r,j}^{s}+\lambda_{j}N_{r,j}^{s}+\varepsilon_{L,j}$$

(3) $$D_{r,j}^{s}=({\dot{\rho }}_{r}^{s}+c({\dot{t}}_{r}-{\dot{t}}^{s})+c({\dot{d}}_{r,j}-{\dot{d}}_{j}^{s})+{\dot{T}}_{r}^{s}+{\dot{I}}_{r,j}^{s}+\varepsilon_{D,j})/\lambda_{j}$$

where the indices *r*, *s*, *j* refer to receiver, GNSS satellite (GPS and BDS), and signal frequency (GPS: L1 and L2, BDS: B1 and B2), respectively; *c* is the light speed in vacuum; *P*, *L*, and *D* denote code, phase, and Doppler observations, respectively; $\rho_{r}^{s}$ is the geometry distance between the antenna phase center of receiver and satellite; *t_r_* and *t^s^* are clock offsets of receiver and satellite; d and b denote the hardware delays on code and phase, where *b* will be absorbed in ambiguity in float PPP [7]; *T* and *I* indicate the tropospheric delay and ionospheric delay; λ and *N* are the wavelength and ambiguity; ε is the sum of observing noises and un-modelled errors; and $\dot{\left(\right)}$ denotes the variations of variables (e.g., geometry distance, clock offset of receiver and satellite, troposphere delay, and ionosphere delay). The troposphere delay, ionosphere delay, and the geometric distance ρ and its variation can be expressed, respectively, as

(4) $$T_{r}^{s}=m_{r,dry}^{s}z_{dry}+m_{r,wet}^{s}z_{wet}$$

(5) $$I_{r}^{s}=40.28\cdot VTEC/(f_{j}^{2}\cdot \cos\vartheta )$$

(6) $$\rho_{r}^{s}=\left\|p_{r,G/B}-p^{s}\right\|\&{\dot{\rho }}_{r}^{s}=\left\|v_{r,G/B}-v^{s}\right\|$$

where *m* and *z* denote the troposphere mapping function (e.g., global mapping function [34]) and the zenith delay (e.g., Saastamoinen model [35]) of the dry (*dry*) and wet (*wet*) components of troposphere delay; *VTEC*, ϑ, and *f* are the vertical total electronic content, zenith angle, and phase frequency, respectively; ***p****_r,G/B_* and ***p****^s^* are the positions at antenna phase center of receiver and satellite; ***v****_r,G/B_* and ***v****^s^* are the corresponding velocities, and ‖ ‖ indicates the modular arithmetic.

For raw PPP, the error terms are either corrected by using empirical models or are estimated as parameters, which is different from the processing method in the conventional PPP mode. Whereas, the earth rotation, relativity, antenna PCO/PCV, wind-up, solid tide, polar migration, dry troposphere delay, and code hardware delays on the satellite side are corrected by classic models and International GNSS Service (IGS) products [33], the receiver clock, code hardware delay of both GPS and BDS on the receiver side, ionosphere delays on frequencies L1 and B1, and ambiguity of each phase observation are estimated as parameters [32]. For the GPS + BDS case, there is inter-system bias (ISB) between GPS and BDS. Therefore, ISB is also estimated as a parameter. Thus, the estimated parameter vector is

(7) $$x={\left[p_{r,G/B},v_{r,G/B},t_{r},{\dot{t}}_{r},z_{wet},b_{B-G},d_{r,j},I_{r,j}^{s},N_{r,j}^{s}\right]}^{T}$$

where $b_{B-G}$ is the ISB of BDS with respect to GPS. Note the fact that there is a strong correlation between the ionosphere delay and code hardware delay. Therefore, a priori ionosphere delay (e.g., by using the Kolubchar model [36] and global ionosphere mapping [37]) is used to separate them as much as possible, and the corresponding function can be expressed as

(8) $$I_{r,j}^{s}=I_{r,priori}^{s}=40.28\cdot VTEC_{priori}/(f_{j}^{2}\cdot \cos\vartheta ),\sigma_{I_{r,priori}^{s}}^{2}$$

where $\sigma_{I_{r,priori}^{s}}^{2}$ is the priori variance of the ionosphere model. Since the ionosphere delay is a frequency dependent error (Equation (5)), $I_{r,2}^{s}$ can be expressed as $I_{r,2}^{s}=I_{r,1}^{s}f_{1}^{2}/f_{2}^{2}$. Therefore, only one ionospheric parameter is estimated for each satellite. Besides, since the receiver hardware delay *d_r_* is correlated to ionospheric delay, it is hard to directly estimate the absolute values for *d_r_*. Thus, only the differential form (i.e., *DCB_r_* = *d_r,_*_1_ − *d_r,_*_2_, *DCB* stands for differential code bias) between *d_r,_*_1_ and *d_r,_*_2_ is estimated. Finally, the state vector can be written as

(9) $$x={\left[p_{r,G/B},v_{r,G/B},t_{r},{\dot{t}}_{r},z_{wet},b_{B-G},DCB_{r,G},DCB_{r,B},I_{r,1}^{s},N_{r,1}^{s},N_{r,2}^{s}\right]}^{T}.$$

Then, the EKF [31] is applied to estimate the parameters. The corresponding state prediction and measurement update functions can be respectively written as

(10) $${\bar{x}}_{k}=\phi_{k/k-1}x_{k-1},{\bar{P}}_{{\bar{x}}_{k}}=\phi_{k/k-1}P_{x_{k-1}}\phi_{k/k-1}^{T}+Q_{k-1}$$

(11) $${\hat{x}}_{k}={\bar{x}}_{k}+K_{k}(Z_{k}-H_{k}{\bar{x}}_{k}),{\hat{P}}_{{\hat{x}}_{k}}=\left(I-K_{k}\right)\left(\phi_{k/k-1}{\bar{P}}_{{\bar{x}}_{k}}\phi_{k/k-1}^{T}+Q_{k-1}\right){\left(I-K_{k}\right)}^{T}+K_{k}R_{k}K_{k}^{T}$$

where $\bar{\left(\right)}$ and $\hat{\left(\right)}$ represent the predicted and updated values; $\phi_{k}$ denotes the state transform matrix from epoch *k*-1 to *k*, which is determined by the state prediction functions, such as constant-velocity model for ***p****_r_* and *t_r_*, white noise procedure for ***v****_r_,t_r_*, and ${\dot{t}}_{r}$, random walk for *z_wet_*, *DCB_r_*, $b_{B-G}$, and $I_{r}^{s}$, and random constant for $N_{r}^{s}$. $K_{k}={\bar{P}}_{{\bar{x}}_{k}}H_{k}^{T}\left(H_{k}{\bar{P}}_{{\bar{x}}_{k}}H_{k}^{T}+R_{k}\right)$ is the gain matrix of EKF; $Q_{k-1}$, $R_{k}$, and **I** denote the state noise variance, innovation ($Z_{k}$) vector’s noise variance, and unit vector, respectively; $H_{k},$ which is the designed matrix obtained by making derivation on Equations (1)–(4) and Equation (8) with respect to Equation (9), can be expressed as

(12) $$H_{k}=\frac{\partial f(P_{r,j}^{s},L_{r,j}^{s},D_{r,j}^{s},T_{r}^{s},I_{r}^{s})}{\partial x}.$$

### 2.2. INS Update

INS can provide navigation solutions by processing measurements from IMU sensors after giving the initial status. As illustrated in Figure 2, the original IMU outputs are the specific force (**f**) and angular rate (ω) vectors, which contain bias (***S***) and scale factor (***B***) errors

(13) $$f^{b}=\left(I+S_{f}\right){\tilde{f}}^{b}+B_{f}\Delta t+\varsigma_{f}$$

(14) $$\omega_{ib}^{b}=\left(I+S_{\omega }\right){\tilde{\omega }}_{ib}^{b}+B_{\omega }\Delta t+\varsigma_{\omega }$$

where ***B***, ***S***, Δt, and $\tilde{\left(\right)}$ are the sensor biases, sensor scale factor, IMU time interval, and theoretical value, respectively; *i* and *b* denote the inertial and body coordinate frames, respectively. ς represents the observing noise. After sensor error correction, motion errors, such as the rotational and sculling on velocity and coning on attitude [25], are also removed by using

(15) $$\Delta v_{Rot+Scu}=\frac{\left(\Delta \theta_{\omega ,k}\times \Delta v_{f,k}^{b}\right)}{2}+\frac{\left(\Delta \theta_{\omega ,k-1}\times \Delta v_{f,k}^{b}+\Delta v_{f,k-1}^{b}\times \Delta \theta_{\omega ,k}\right)}{12}$$

(16) $$\Delta \theta_{Con}=\frac{\Delta \theta_{\omega ,k-1}\times \Delta \theta_{\omega ,k}}{12}$$

with

(17) $$\Delta v_{f}=\int_{k-1}^{k}fdt,\Delta \theta_{\omega }=\int_{k-1}^{k}\omega_{ib}^{b}dt$$

where $\Delta \theta_{k}$ and $\Delta v_{k}$ are the attitude and velocity increments at epoch *k*, and × refers to the cross-product computation. After corrections from Equation (14) to (17), the INS updated position (Equation (18)), velocity (Equation (19)), and attitude (Equation (20)) in the navigation frame (*n*) can be obtained by

(18) $$p_{r,INS,k}^{n}=\left(\begin{array}{c} B\\ L\\ H \end{array}\right)=\left(\begin{array}{c} -2\cdot \mathrm{atan}\left(q_{n}^{e}\left[2\right]/q_{n}^{e}\left[0\right]\right)-0.5\pi \\ 2\cdot \mathrm{atan}\left(q_{n}^{e}\left[3\right]/q_{n}^{e}\left[0\right]\right)\\ H_{k-1}-v_{D,mid}\cdot \Delta t \end{array}\right)$$

(19) $$v_{r,INS,k}^{n}=v_{r,INS,k-1}^{n}+\int_{k-1}^{k}[C_{b}^{n}f^{b}-\left(2\omega_{ie}^{n}+\omega_{en}^{n}\right)\times v_{r,INS,k-1}^{n}+g^{n}]dt$$

(20) $$\left(\begin{array}{c} \gamma \\ \varphi \\ \psi \end{array}\right)=\left(\begin{array}{c} \mathrm{atan}\left(-C_{b}^{n}[3,1]/\sqrt{C_{b}^{n}{\left[3,2\right]}^{2}+C_{b}^{n}{\left[3,3\right]}^{2}}\right)\\ \mathrm{atan}2\left(C_{b}^{n}\left[3,2\right],C_{b}^{n}\left[3,3\right]\right)\\ \pi +\mathrm{atan}2\left(C_{b}^{n}\left[2,3\right]+C_{b}^{n}\left[1,2\right],C_{b}^{n}\left[1,3\right]+C_{b}^{n}\left[2,2\right]\right) \end{array}\right)$$

where B, *L*, and *H* are geodetic latitude, longitude, and height; $v_{D,mid}$ is the velocity at middle time between epochs *k*-1 and *k*; *e* is the earth-fixed-earth-centered coordinate frame; $\omega_{AD}^{C}$ refers to the rotation angular rate of *A* (e.g., *i*, *e*, and *n*) frame with respect to *D* frame (e.g., *i*, *e*, and *n*), projected in *C* frame (e.g., *i*, *e*, and *n*); $g^{n}$ and ⨂ stand for the normal gravity vector at $p_{r,k}^{n}$ and the quaternion product; and $q_{n}^{e}\left[k1\right]$ and $C_{b}^{n}\left[k2,k3\right]$ denote the *k*1 element in quaternion $q_{n}^{e}$ and the *k*2-row *k*3-column in the direction cosine matrix $C_{b}^{n}$.

### 2.3. Robust Extended Kalman Filter Based PPP/INS Tight Integration

As mentioned above, the positions provided by INS are related to the initial status. Therefore, in order to get high-accuracy position solutions, a high-accuracy initial position is needed. Here, the positions calculated by PPP in Equation (12) are utilized for position/velocity initialization and the static IMU data are adopted for attitude initialization [20]. As depicted in Figure 2, time synchronization between GPS/BDS observations and INS solutions is applied after the INS update. When both GPS/BDS and INS data are available, the tight integration function works. To compensate for the lever-arm (system offset) between INS and GPS/BDS, the INS position and velocity updates are transformed to the GPS/BDS receiver antenna phase center [20] by using

(21) $$p_{r,G/B}^{n}=p_{r,INS}^{n}+ℜC_{b}^{n}l^{b}$$

(22) $$v_{r,G/B}=v_{r,INS}-\left(\omega_{ie}^{n}\times +\omega_{en}^{n}\times \right)C_{b}^{n}l^{b}-C_{b}^{n}\left(l^{b}\times \right)\omega_{ib}^{b}$$

where $l^{b}$ is the lever-arm of the GPS/BDS receiver antenna phase center with respect to the IMU center, which is measured accurately before data collection; $ℜ=\mathrm{diag}\left(\frac{1}{R_{M}+H},\frac{1}{\left(R_{N}+H\right)cosB},-1\right)$ denotes the matrix to transform the lever-arm from n-frame to *e*-frame. The transformed position and velocity can be used together with the precise orbit/clock products to predict code (***P****_INS_*), phase (***L****_INS_*), and Doppler (***D****_INS_*). Then, these predicted values and the observed data can be used to form the innovation vector of EKF for PPP/INS tight integration as

(23) $$Z_{k}=\left(\begin{array}{c} P_{j}-P_{INS,j}\\ L_{j}-L_{INS,j}\\ D_{j}-D_{INS,j}\\ I_{1}-I_{priori,1} \end{array}\right),\begin{array}{c} GPS:j=L1\&L2\\ BDS:j=B1\&B2 \end{array}.$$

The corresponding state parameter vector, which contains both PPP relative parameters and INS relative parameters, can be written as

(24) $$x={\left[p_{r,G/B},v_{r,G/B},\theta ,B_{f},S_{f},B_{\omega },S_{\omega },t_{r},{\dot{t}}_{r},z_{wet},b_{B-G},DCB_{r,G},DCB_{r,B},I_{r,1}^{s},N_{r,1}^{s},N_{r,2}^{s}\right]}^{T}$$

where the symbols are the same as those mentioned above. The design matrix can be obtained by making derivation on Equation (23) with respect to Equation (24) (i.e., $\partial Z_{k}/\partial x$). The state transform matrix ϕ for PPP/INS tight integration is different from that used in Equation (10). In general, the PSI-angle model [25], instead of the constant velocity model and white noise model, is adopted to describe the behavior of position, velocity, and attitude; the first-order Gauss–Markov procedure [25] is used to indicate variations of IMU biases and scale factors. The other state functions remain the same as those in PPP. Then, by applying EKF [31,38] as described in Equations (10) and (11), the integration solutions can be obtained.

However, as the qualities of BDS GEO and IGSO are different, it is necessary to increase the benefit from IGSO and decrease the limitation of GEO. In this paper, the R-EKF [31] is adopted to detect the low quality observations and reduce the weight (e.g., by enlarging the variance). Thus, the equivalent weight function [39] instead of the empirical weight function [6] is used by

(25) $${\tilde{R}}_{k}=\frac{1}{\alpha }R_{k}=\alpha P_{k}^{-1}$$

where ${\tilde{R}}_{k}$ and α refer to the equivalent variance matrix and the robust-factor matrix. Then α is calculated by using the famous weight function [39]

(26) $$\alpha_{m}=\{\begin{array}{c} \begin{array}{cc} \begin{array}{ccc} \begin{array}{cc} \begin{array}{cc} 1.0 & \end{array} & \end{array} & & \end{array} & \left|{\overset{⌣}{V}}_{m}\right|\leq c_{0} \end{array}\\ \begin{array}{cc} \frac{c_{0}\left(c_{1}-\left|{\overset{⌣}{V}}_{m}\right|\right)}{c_{1}-c_{0}} & c_{0}<\left|{\overset{⌣}{V}}_{m}\right|\leq c_{1} \end{array}\\ \begin{array}{cc} \begin{array}{cccc} \begin{array}{cc} 0.0 & \end{array} & & & \end{array} & \left|{\overset{⌣}{V}}_{m}\right|\geq c_{1} \end{array} \end{array}$$

where $c_{0}$ and $c_{1}$ are constants with empirical values of $c_{0}$ in 1.0 to 1.5 and $c_{1}$ in 2.5 to 8.0 [39]. $c_{0}=$ 1.0 and $c_{1}$ = 2.5 are used in the data processing. $\left|{\overset{ˇ}{V}}_{m}\right|=\left|V_{m}\right|/\sigma_{V_{k}}$ is the standardized *m*^th^ posteriori observation residual ($V_{k}$) and $\sigma_{V_{k}}$ is the variance of $V_{k}$. Therefore, the posteriori observation residuals can be obtained by

(27) $$V_{k}=H_{k}{\hat{x}}_{k}-Z_{k}$$

where symbols are the same as those mentioned above.

As shown in Equation (27), the residuals from top to bottom will be divided into good quality, low quality, and gross, respectively. The weight of gross observations is set to zero; however, it would cause the equivalent variance matrix to be a singular matrix. Therefore, a pimping value is used in the data processing. Then, the robust estimation can be obtained by using the robust gain matrix

(28) $${\tilde{\hat{x}}}_{k}={\bar{x}}_{k}+{\tilde{K}}_{k}(Z_{k}-H_{k}{\bar{x}}_{k})$$

(29) $${\tilde{K}}_{k}={\bar{P}}_{{\bar{x}}_{k}}H_{k}^{T}{\left(H_{k}{\bar{P}}_{{\bar{x}}_{k}}H_{k}^{T}+{\tilde{R}}_{k}\right)}^{-1}$$

where $\tilde{\hat{\left(\;\right)}}$ denotes the robust values, and the other symbols are the same as those mentioned above.

## 3. Tests, Results, and Discussion

**Figure 3:**
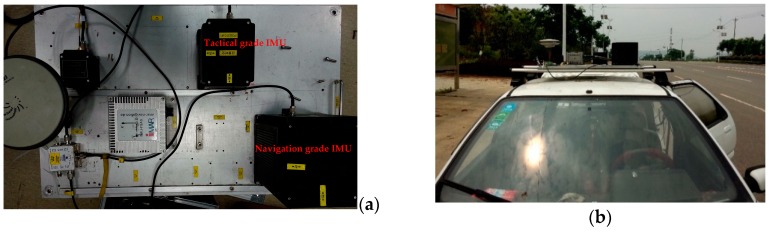
IMUs and GPS/BDS antenna (**a**) used in the test and their locations on the land-borne vehicle (**b**).

A land-borne vehicle test, which used a GPS + BDS receiver and two IMUs (i.e., a tactical grade IMU and a navigation grade IMU), was conducted in the area around Wuhan, China to collect 1 Hz GPS + BDS data and 200 Hz IMU data. In order to ensure that the IMU measurements could reflect the real vehicle motion, both IMUs and the GPS/BDS receiver antenna were fixed rigidly on a steel plate (as shown in Figure 3a), and the steel plate was fixed on the top of the vehicle. In this case, the lever-arm of the receiver antenna with respect to the two IMU sensors could be measured accurately to the millimeter-level (as listed in Table 1). Such measured lever-arms were used in Equations (21) and (22) to transform the INS predicted position and velocity from the IMU center to the GPS/BDS receiver antenna phase center. The steel plate was fixed on the top of vehicle (as shown in Figure 3b). In the data collecting phase, the vehicle was kept static for about five minutes. The data during this period was used to provide the initial status (e.g., position, velocity, and attitude) for the tight integration.

### 3.1. Test Description

**Figure 4:**
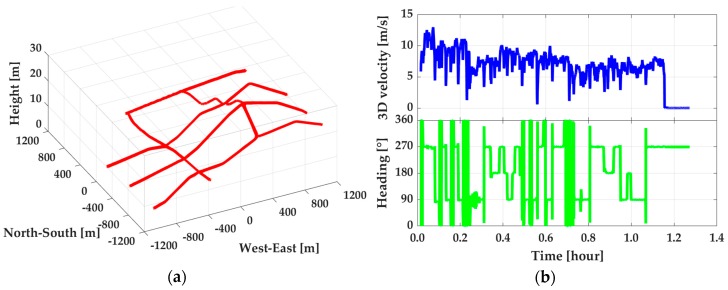
Trajectory in navigation frame (**a**) and the 3D velocity and movement direction (**b**) of the vehicle.

**Figure 5:**
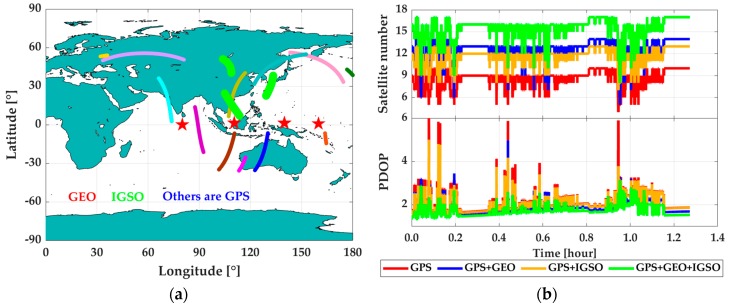
Satellite sky plot (**a**) and the corresponding position dilution of precision (PDOP) (**b**) during the land-borne test.

The test area was around 1200 × 1200 m^2^ large and was approximately 20 kilometers away from the reference station. The whole test lasted for about 1.4 h, as shown in Figure 4. With such a short base-line, the smoothed solutions from GPS + BDS RTK/INS (with use of the navigation grade IMU) tight integration were used as the reference values to validate the proposed method. During the test, the vehicle moved approximately along the directions of south-north and east-west with a speed of within 13 m/s. The heading angle changed periodically around 0, 90, 180, 270, and 360°, which led to the ‘#’ shape route of the mission. The undulant test area could improve the observability for gyro error estimation and further improve the performance of PPP/INS tight integration. Shown in Figure 5 is the visible satellite number during the mission. From Figure 5a, it is clear that 12 GPS satellites and 7 BDS satellites were observed (including 3 IGSO satellites (C7, C8, C10) and 4 GEO satellites (C1, C2, C3, C4)). The visible satellite number under different combinations and the corresponding position dilution of precision (PDOP) value were plotted in Figure 5b. From the statistics, the average visible satellite numbers were 8.6, 12.4, 11.6, and 15.4 for GPS, GPS + GEO, GPS + IGSO, and GPS + GEO + IGSO, respectively, and the corresponding PDOP were 2.1, 1.9, 2.0, and 1.7. Therefore, the use of both GEO and IGSO can enhance GPS’ observing condition and the corresponding geometry structure.

In the data processing, both PPP and PPP/INS tight integration modes were adopted and the EKF and R-EKF were used as the estimators. The un-differenced and un-combined phase and code observations from GPS on L1 and L2 frequencies and BDS on B1 and B2 frequencies were processed by setting the cut of elevation angle to 10°. The final precise satellite products of 300s-orbit and 30s-clock of multi-GNSS from German Research Centre for Geosciences (GFZ) were adopted. The elevation dependent weight function [6] and the equivalent weight function [39] in Equation (26) were used in normal- and robust-EKF. In the analysis phase, the first five-minute solutions were excluded because of the convergence time in both PPP and PPP/INS tight integration.

### 3.2. Performance Analysis

**Figure 6:**
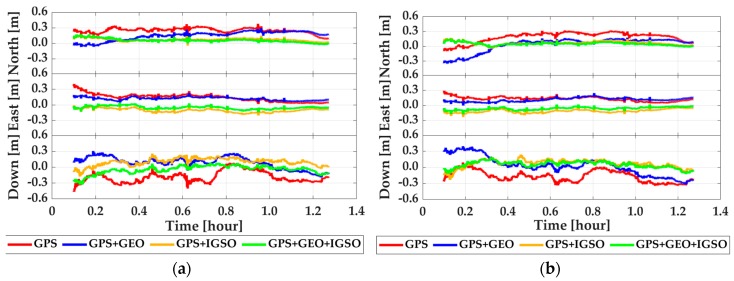
Position offsets of PPP (**a**) and PPP/INS tight integration (**b**) by comparing with GPS + BDS RTK/INS tight integration solutions.

**Figure 7:**
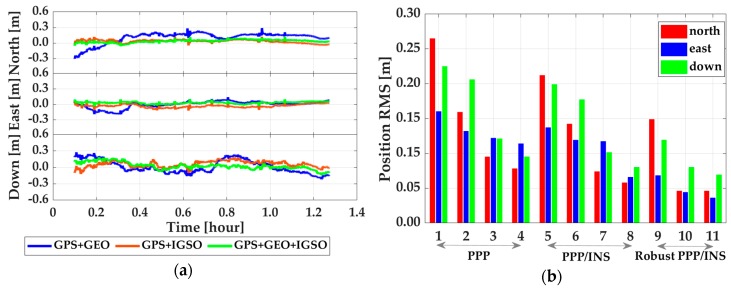
Position offsets of robust PPP/INS tight integration (**a**) and position RMS of all data processing modes (**b**); in subfigure (**b**), 1–4 denote PPP using GPS, GPS + GEO, GPS + IGSO, and GPS + GEO + IGSO, 5–8 denote PPP/INS tight integration using GPS, GPS + GEO, GPS + IGSO, and GPS + GEO + IGSO, 9–10 denote robust PPP/INS tight integration using GPS + GEO, GPS + IGSO, and GPS + GEO + IGSO.

**Figure 8:**
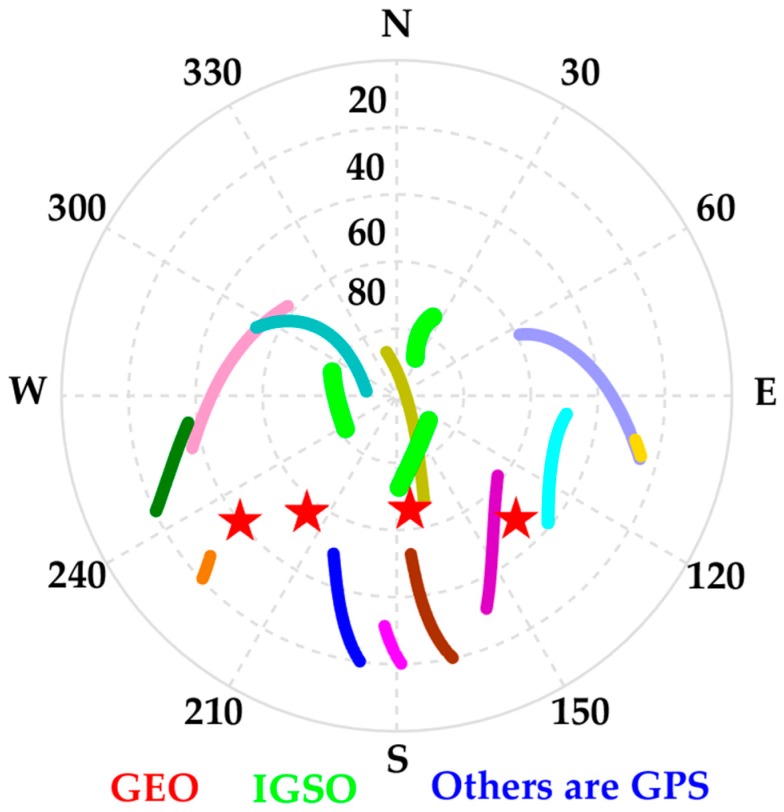
Azimuth-elevation based sky plot of the observed GEO, IGSO, and GEO satellite.

Shown in Figure 6 and Figure 7 are the time-series of position offsets in n-frame (i.e., north–east–down), which were obtained by making differences between PPP, EKF based PPP/INS tight integration, and R-EKF based PPP/INS tight integration using GPS, GPS + GEO, GPS+IGSO, and GPS + GEO + IGSO data with the reference values. According to the statistics in Figure 6a, the positioning accuracy of GPS PPP could be upgraded while using GPS + GEO and GPS + IGSO. While using both GEO and IGSO together, the position offsets reduced even more. According to the Root Mean Square (RMS) values listed in Table 2, the position RMSs were upgraded from 0.265, 0.160, and 0.225 m in GPS PPP mode to 0.078, 0.114, and 0.095 m in GPS + GEO + IGSO PPP mode, with improvements of 70.5%, 28.7%, and 57.8% in the north, east, and down directions, respectively. The corresponding accuracy enhancements were about 40.0%, 17.5%, and 8.4% by adding GEO and 64.2%, 23.7%, and 46.2% by adding IGSO. This means that the IGSO satellites provided more enhancement than GEO, which may be due to the almost static locations of GEO satellites over the equator. As shown in Figure 8, all the four observed GEO satellites were almost in a straight line in the south, while the IGSO satellites moved around the mission area. Such a constellation means that IGSO can provide much more user-satellite geometry structure improvements than GEO. Besides, a small mutation in the north component of GPS PPP emerged at around 0.95 h, which may be because there were only four visible GPS satellites (as shown in Figure 5b). However, such mutations were rather small when no cycle slips occurred because the receiver DCB, ionosphere delays, and troposphere delay should have been constants in the short term, which could have provided strong constraints for PPP calculations.

According to the RMS results in Figure 7b and Table 2, both INS and R-EKF presented positive influences on PPP position accuracy. For the PPP/INS tight integration case, the average improvements from INS were 19.6%, 17.6%, and 14.5% in the three directions compared to the PPP solutions. The enhancements from GEO and IGSO were 33.0%–13.1%–11.1% and 65.1%–14.6%–49.2% in north–east–down components. For the R-EKF based PPP/INS tight integration case, the average enhancements from R-EKF were 17.8%, 50.2%, and 22.4% compared to the solutions from EKF, and 32.9%, 60.3%, and 34.5% compared to PPP solutions. Visibly, both INS and R-EKF could make the solutions more stable and accurate.

**Figure 9:**
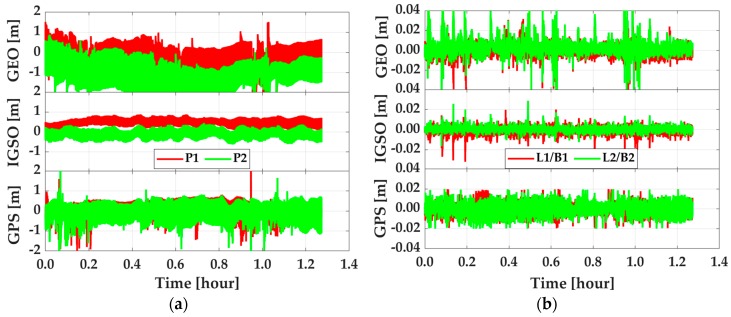
Code residuals (**a**) and phase residuals (**b**) of GEO, IGSO, and GPS satellites.

The contribution of INS to enhancing PPP was largely due to its low-pass filter characteristics and the enhanced observability in the vertical direction, which was been presented in [10,20,24,40,41]. Although the enhancement from BDS on GPS has been proven by previous works [11,13,14], the effect of different constellations of BDS satellites on GPS has not been demonstrated. Most of those works only adopted the empirical priori weight and the satellite elevation dependent weight function [6] to decrease GEO’s influence. However, observation’s real quality would change along with users’ environments, especially in the dynamic cases. Then, the empirical method may not work well in decreasing the influence of GEO satellites. However, the real quality of GPS and BDS can be reflected directly by the posteriori residuals that are obtained from Equation (26). As plotted in Figure 9, the posteriori residuals of codes and phases of GEO, IGSO, and GPS were significantly different from each other. According to the statistics listed in Table 3, the quality of GEO codes in terms of RMS are 0.759 m and 1.406 m for B1 and B2 frequencies, which are about 1.5–4.5 times worse than that of IGSO (0.491 m and 0.292 m) and GPS (0.307 m and 0.424 m). GEO’ phase residual RMS were 5 mm and 7 mm for B1 and B2 frequencies, which was about 1.0–1.5 times lower than IGSO (4 mm and 3 mm) and GPS (3 mm and 4 mm). As such the quality of IGSO was similar to that of GPS. The average variance of posteriori residuals of P1, P2, L1, and L2 of GPS and BDS were 0.004, 0.005, 0.494, and 0.731 m, respectively. By using Equation (26), the re-determined equivalent weight could reflect the real quality of GEO/IGSO/GPS. Then, based on such equivalent weight [39], the R-EKF could increase the weight of GPS and IGSO and decrease that of GEO. Then, the robust positioning solutions was obtained, which can be seen in Figure 7a.

**Figure 10:**
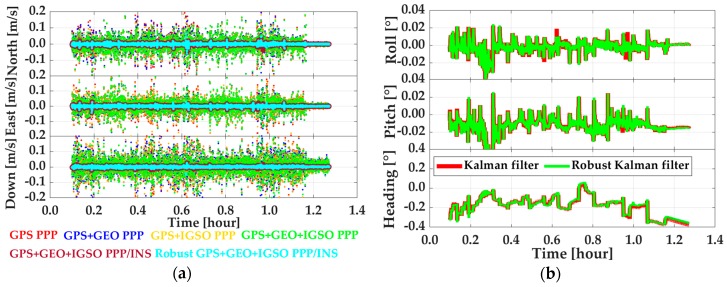
Velocity offsets (**a**) of PPP, PPP/INS, and robust PPP/INS tight integration models, and attitude offsets (**b**) of PPP/INS with and without R-EKF.

**Figure 11:**
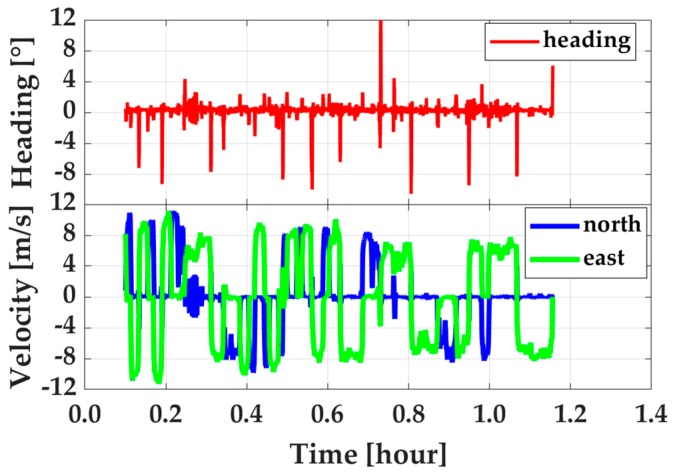
Heading angles calculated by velocity (**top**) from PPP and the corresponding velocity in horizontal (**bottom**).

Besides the position, the velocity and attitude are also important for dynamic users. Therefore, the impacts of GEO, IGSO, INS, and R-EKF on velocity and attitude determination were accessed. In this part, the reference values for velocity and attitude were calculated from RTK/INS tight integration by using navigation grade IMU measurements. Shown in Figure 10 are velocity offsets from PPP, EKF based PPP/INS tight integration, and R-EKF based PPP/INS tight integration and attitude offsets from PPP/INS tight integration with and without robust EKF by using GPS, GPS + GEO, GPS + IGSO, and GPS + GEO + IGSO data, respectively. From Figure 10, it can be seen that INS could enhance velocity accuracy visibly. However, the improvements from GEO, IGSO, and even GEO + IGSO were not significant. This outcome was because the velocity accuracy in PPP mode was mainly dependent on the quality of Doppler measurements (about decimeter per second level), and had little relationship with positioning accuracy [26]. As listed in Table 4, the velocity RMS on average were 4.4 ± 0.4 cm/s, 4.1 ± 0.2 cm/s, and 5.9 ± 0.5 cm/s in north, east, and vertical directions, respectively, in PPP mode. While applying INS, the effect of Doppler noises on velocity was decreased significantly. Then, velocity accuracy was up to around 4 mm/s in all three directions. In this case, the effects of GEO and IGSO on improving velocity were covered by INS, and no improvement could be seen when using GPS + BDS together compared with using only the GPS data. This was because the velocity accuracy in PPP/INS tight integration only depended on the IMU performance [26]. Meanwhile, from Figure 10a and the statistics in Table 4, it can be seen that the R-EKF also had slight effect on velocity. Similarly, works in [26] have also proven that attitude determination accuracy had a weak relationship to GNSS data but was highly determined by IMU sensors’ level. The results in this test also indicated the same conclusion. From the statistics in Table 5, the attitude error RMS on average were about 0.007°, 0.015°, and 0.202° for roll, pitch, and heading, respectively. Compared to roll and pitch, poorer accuracy in heading direction was due to the weaker observability in IMU’s vertical (z-axis) gyroscope [42] and the inadequate maneuver motions (as shown in Figure 4). In contrast, the heading computed by PPP velocity using $heading=\tan^{-1}\left(v_{E}/v_{N}\right)$ [25] was much worse than that of PPP/INS integration, as is shown in Figure 11. As shown, such heading angles were sensitive to horizontal velocity. Therefore, the accuracy of the heading changed regularly along with the velocity, especially when the velocity direction was changed. Such phenomenon can also be found by making a comparation between Figure 11 and Figure 4b.

According to the results above, it can be concluded that GEO and IGSO have different influences on improving PPP performance and the R-EKF that is based on posteriori residuals can effectively adjust the optimal weight for GPS + BDS data processing. Then, more reliable and accurate positioning solutions are provided. However, there is little relationship between these methods and the accuracy of velocimetry and attitude determination.

## 4. Conclusions

This paper presented the method to adopt the posteriori residuals based robust extended Kalman filter to re-determine the weight of BDS GEO and IGSO in GPS + BDS PPP/INS tight integration. The corresponding mathematical models were described and validated by a set of land-borne vehicle tests. Results indicate that (1) The enhancements from BDS on improving PPP performance was significant; however, such enhancements from GEO and IGSO were different. Such appearance was mainly caused by the lower quality and the worse spatial distribution of GEO satellites. (2) The position improvements from IGSO were more visible than those from GEO. However, such improvements were different in different data processing modes. On average, there were about 22.7%, 23.9%, and 7.0% in PPP, EKF based PPP/INS tight integration, and robust extended Kalman filter based PPP/INS tight integration, respectively. When the robust method was utilized, the difference of the influences from GEO and IGSO was narrowed significantly. (3) INS can help to provide high-accuracy velocity and attitude; however, whether using the multi-GNSS or adopting the robust estimator, there are little enhancements on the accuracy of velocity and attitude determination.

From this research, it can be concluded that the robust extended Kalman filter can be applied to BDS related data processing to remove adverse influences from GEO. However, such weaknesses would be reduced along with large progress in improving the signal quality and orbit accuracy of BDS GEO satellites in the future. Particularly, under the BDS-3 case after December 27, 2018, more MEO satellites and better special signal quality will be provided, which will weaken the influence of GEO in data processing and provide better solutions.

## Figures and Tables

**Table 1:** Parameters of IMU sensors and lever-arm of receiver antenna with respect to IMU sensors.

IMU Sensors	Gyro Bias (°/h)	Accelerometer Bias (mGal)	Forward (mm)	Right (mm)	Down (mm)
Navigation Grade	0.001	25	−122.0	613.1	−109.0
Tactical Grade	0.5	500	114.0	429.0	−151.5

**Table 2:** RMS of position offsets of PPP, PPP/INS tight integration (PPP/INS), and robust PPP/INS tight integration (robust PPP/INS) by comparing with GPS + BDS RTK/INS tight integration solutions (unit: m).

Data	PPP			PPP/INS			Robust PPP/INS		
	North	East	Down	North	East	Down	North	East	Down
GPS	0.265	0.160	0.225	0.212	0.137	0.199	-	-	-
GPS + GEO	0.159	0.132	0.206	0.142	0.119	0.177	0.149	0.068	0.119
GPS + IGSO	0.095	0.122	0.121	0.074	0.117	0.101	0.046	0.044	0.080
GPS + GEO + IGSO	0.078	0.114	0.095	0.058	0.066	0.080	0.046	0.036	0.069

**Table 3:** RMS of residuals of codes (P1 and P2) and phases (GPS: L1 and L2, BDS: B1 and B2) of GEO, IGSO, GPS.

Residuals	P1(m)	P2(m)	L1/B1(m)	L2/B2(m)
GEO	0.759	1.406	0.005	0.007
IGSO	0.491	0.292	0.004	0.003
GPS	0.307	0.424	0.003	0.004

**Table 4:** Velocity RMS of PPP, PPP/INS tight integration, and robust PPP/INS tight integration (unit: m).

Data	PPP			PPP/INS			Robust PPP/INS		
	North	East	Down	North	East	Down	North	East	Down
GPS	0.0485	0.0438	0.0629	0.0044	0.0040	0.0039	-	-	-
GPS + GEO	0.0449	0.0399	0.0619	0.0043	0.0040	0.0038	0.0043	0.0040	0.0038
GPS + IGSO	0.0430	0.0418	0.0589	0.0042	0.0040	0.0036	0.0042	0.0040	0.0036
GPS + GEO + IGSO	0.0403	0.0392	0.0533	0.0040	0.0040	0.0036	0.0040	0.0040	0.0036

**Table 5:** Attitude RMS of PPP/INS tight integration with and without robust extended Kalman filter (unit: ^o^).

Data	PPP/INS			Robust PPP/INS		
	Roll	Pith	Heading	Roll	Pith	Heading
GPS + GEO	0.0065	0.0154	0.2044	0.0065	0.0154	0.2030
GPS + IGSO	0.0065	0.0153	0.2028	0.0065	0.0152	0.2023
GPS + GEO + IGSO	0.0065	0.0153	0.2025	0.0064	0.0152	0.1983
